# Potential and Limitations of Cross-Protective Vaccine against Malaria by Blood-Stage Naturally Attenuated Parasite

**DOI:** 10.3390/vaccines8030375

**Published:** 2020-07-11

**Authors:** Takashi Imai, Kazutomo Suzue, Ha Ngo-Thanh, Chikako Shimokawa, Hajime Hisaeda

**Affiliations:** 1 Department of Infectious Diseases and Host Defense, Gunma University Graduate School of Medicine, Maebashi, Gunma 371-8511, Japan; suzue@gunma-u.ac.jp (K.S.); dr.ngothanhha@gmail.com (H.N.-T.); 2 Department of Parasitology, Graduate School of Medical Sciences, Kyushu University, Fukuoka 812-8582, Japan; 3 Department of Parasitology, National Institute of Infectious Diseases, Tokyo 162-0052, Japan; chikakos@nih.go.jp (C.S.); hisa@nih.go.jp (H.H.)

**Keywords:** Malaria, live vaccine, whole parasite, spleen, lymph node, *Plasmodium*, *Plasmodium yoelii*, *Plasmodium berghei*

## Abstract

Human malaria vaccine trials have revealed vaccine efficacy but improvement is still needed. In this study, we aimed to re-evaluate vaccination with blood-stage naturally attenuated parasites, as a whole-organism vaccine model against cross-strain and cross-species malaria, to establish a better vaccination strategy. C57BL/6 mice controlled blood-stage *Plasmodium yoelii* 17XNL (PyNL) within 1 month of infection, while mice with a variety of immunodeficiencies demonstrated different susceptibilities to PyNL, including succumbing to hyperparasitemia. However, after recovery, survivors had complete protection against a challenge with the lethal strain PyL. Unlike cross-strain protection, PyNL-recovered mice failed to induce sterile immunity against *Plasmodium berghei* ANKA, although prolonged survival was observed in some vaccinated mice. Splenomegaly is a typical characteristic of malaria; the splenic structure became reorganized to prioritize extra-medullary hematopoiesis and to eliminate parasites. We also found that the peritoneal lymph node was enlarged, containing activated/memory phenotype cells that did not confer protection against PyL challenge. Hemozoins remained in the spleen several months after PyNL infection. Generation of an attenuated human blood-stage parasite expressing proteins from multiple species of malaria would greatly improve anti-malaria vaccination.

## 1. Introduction

Malaria is a life-threatening infectious disease with a very long history of human infection. We can read of characteristic malaria symptoms, splenomegaly and periodic fatal fever in ancient writing from 2700 BC in China and Egypt [1]. Around 5000 years has passed, but this deadly parasite still coexists with humans. Following the emergence of anti-malaria drug-resistant parasites, anti-insecticide-resistant mosquitoes [2,3], and the recently emerged multidrug-resistant *Plasmodium falciparum* (Pf) in Cambodia, which then spread to Thailand and Vietnam, this super Pf has acquired both artemisinin and piperaquine resistance [4]. Thus, malaria vaccine development is a high priority in endemic areas and is also of great benefit for travelers from malaria-free countries [5].

Many vaccine trials are now in the clinical study phase for human malaria [5,6,7,8,9,10], such as a subunit vaccine, which uses part of a protein that is an important factor for eliciting antibody responses or induction of T cell mediated immunity [10]. The malaria vaccine closest to being ready for commercial distribution is RTS,S/AS01 (Mosquirix, GlaxoSmithKline) [11,12,13]. This vaccine contains a portion of the Pf circumsporozoite protein (CSP) along with hepatitis B virus envelope protein and chemical adjuvant, targeting the sporozoite (a stage of the malaria parasite released by infected mosquitoes) or liver-stage parasites (sporozoites infect hepatocytes and develop daughter cells for the subsequent blood-stage of infection) [13]. Regarding vaccine trial results of RTS,S [14,15], the WHO stated ‘The Phase 3 clinical trial for RTS,S demonstrated efficacy of the vaccine when given to children aged 5–17 months, resulting in significant reductions in malarial disease and associated hospitalizations [16]. This vaccine was subsequently launched in 2019 in Malawi, Ghana, and Kenya for 360,000 children per year in selected endemic areas [16]. This trial will show the efficacy in large-scale administration.

Similarly, blood-stage subunit malaria vaccines have also been developed for clinical use. BK-SE36 is a candidate subunit vaccine for blood-stage Pf that uses a portion of the SERA protein, which is a surface antigen present on parasitized red blood cells (pRBC). The protective efficacy of BK-SE36 was 72% after 1 year of administration for people aged 6–20 years in Uganda [17].

Another type of vaccine is the whole organism vaccine [10]. Vaccination with whole irradiated sporozoites is one of the most effective vaccines against challenge with homologous parasite strains, yielding up to 100% sterile protection [18,19]. However, irradiated sporozoites are not as effective against heterologous parasite strains [20,21]. Genetically attenuated malaria parasite (GAP) is another candidate for a liver-stage vaccine. Schajik and colleagues have developed the GAP-Pf, which lacks the b9 and slarp genes in the sporozoite stage [22]. This vaccine candidate was used in controlled human malaria infection in a Phase 1/2a trial. While the safety profile and induction of a Pf specific immunological response was confirmed, the expected strong efficacy was not apparent in this trial [23].

Vaccines targeting liver-stage parasites have the inherent problem; if the vaccine allows even one mature merozoite to emerge from the liver into the blood, parasite replication will occur unchecked. However, some antigens are common between liver- and blood-stage parasites [24]. In contrast to liver-stage vaccines, we do not have a blood-stage GAP for use in humans. However, there are some benefits for using whole organism vaccines. For example, whole organism vaccines contain the full complement of parasite proteins, while subunit vaccines usually contain only one or several target proteins. Therefore, if the dominant malaria parasite in an endemic area acquires a mutation in the gene targeted by the subunit vaccine, the vaccine is likely to be rendered ineffective, while a whole-organism vaccine will be more stable against single gene mutations.

Our group has studied protective immunity and pathology in blood-stage malaria [25,26,27,28,29]. In this study, we aimed to model the blood-stage human malaria parasite in individuals in malaria non-endemic areas using the mouse blood-stage malaria infection model. We re-evaluated the value of the whole blood-stage parasite vaccine, particularly in terms of administration to immunodeficient animals, cross protective immunity, and side effects. We also investigated effects in the spleen, which is an important organ for protection against blood-stage malaria [30,31]. We conclude that cross-strain vaccination is possible but cross-species vaccination is difficult, and such protection is dependent on the spleen.

## 2. Materials and Methods

### 2.1. Animals

Male and female C57BL/6 wild type (WT) mice, C57BL/6JSlc-Gld (Gld: generalized lymphoproliferative disease; FasL mutant) mice, and C57BL/6JSlc-Lpr (Lpr: Fas^lpr^) mice were obtained from SLC (Hamamatsu, Japan) or Kyudo (Tosu, Japan). Perforin knock out (KO) mice and IFN-gamma receptor KO mice were obtained from Jackson Immuno Research Laboratories (West Grove, PA). Immunoproteasome subunit LMP7 KO mice [32] on the B6 background were kindly provided by Dr. Fehling (University Clinics Ulm, Ulm, Germany). Milk fat globule-EGF factor 8 protein (MFG-E8) KO mice [33] were kindly provided by Dr. Hanayama (Kanazawa University, Kanazawa, Japan) IL-17A KO mice [34] were kindly provided by Dr. Iwakura (University of Tokyo, Tokyo, Japan). All mice were maintained under specific-pathogen-free conditions. Experiments were performed in mice aged 8–16 weeks. No significant differences were observed between male and female mice in our experiments.

All animal experiments were approved by the Committee for Ethics on Animal Experiments of the Faculty of Medicine and adhered to the Guidelines for Animal Experiments of the Faculty of Medicine, Kyushu University and Gunma University, Japan, under Japanese law (no. 105) and notification (no. 6) of the Government of Japan. All experiments were performed in accordance with relevant guidelines and regulations.

### 2.2. Parasites and Infection

The blood-stage non-lethal strain *Plasmodium yoelii* 17XNL (PyNL) and lethal strain *P. yoelii* 17XL (PyL), and the uncloned line of *Plasmodium berghei* ANKA (PbA) were generous gifts from Dr M Torii (Ehime University, Matsuyama, Japan), and stored in liquid nitrogen. Red blood cells (RBCs) parasitized by each strain (pRBCs) were prepared in donor WT mice injected intraperitoneally (i.p.) with parasite stock and stored in liquid nitrogen. The mice were infected by i.p. injection with 50,000 pRBCs suspended in RPMI1640 (sigma) from donor mice. Giemsa-staining of blood-smears was used to determine parasitemia.

### 2.3. Anti-Malaria Drug Treatment

Artesunate (Art; Sigma, St. Louis, MO) is a commonly used anti-malaria drug [35]. Art was dissolved in saline (Otsuka Pharmaceutical, Tokyo, Japan) containing 5% NaHCO_3_ and administered i.p. for 5 consecutive days (64 mg/kg body weight/day).

### 2.4. Histology

The mice were sacrificed, spleens were collected and fixed with 4% paraformaldehyde, then samples were embedded in paraffin wax. Sections of 2.5 µm thickness were obtained and mounted on glass slides. Hematoxylin and eosin (H&E) staining was performed on the sections. For BAND3 staining, rabbit-polyclonal anti-BAND3 (Abcam) and Alexa Fluor 532 goat anti-rabbit IgG (H + L, Thermo Fisher Scientific) and DAPI (Sigma-Aldrich) was used. Organ sectioning and staining was performed by Biopathology Institute Co., Ltd. (Oita, Japan). The slides for histology were analyzed and images were taken using BZ-II software and a Biorevo BZ-9000 microscope (Keyence, Osaka, Japan).

### 2.5. Flow Cytometry

Cell suspensions from spleen and lymph node (LN) without RBC lysis were incubated with anti-CD16/32 antibody (clone: 2.4G2) for Fc blocking and stained with following fluorochrome-labeled antibodies. AF488-conjugated anti-CD4 (clone: GK1.5), PE-Cy7-conjugated anti-CD8a (clone: 53-6.7), PE-conjugated anti-CD3 (clone: 17A2), APC-conjugated anti-TER119 (clone: Ter119), FITC-conjugated anti-CD44 (clone: IM7), PE-conjugated anti-B220 (clone: RA3-6B2), PE-conjugated anti-CD11b (clone: M1/70), PE-Cy7-conjugated anti-F4/80 (clone: BM8.1) and a Zombie NIR™ Fixable Viability Kit (Biolegend, San Diego, CA) were used for live cell/dead cell discrimination according to manufacturer’s protocol. Isotype control antibodies were also used to evaluate specific staining. Cells were analyzed with a FACSCalibur, FACSVerse flow cytometer (Becton Dickinson, San Jose, CA), and data were analyzed with FlowJo software (Treestar, Ashland, OR). The gating strategy is shown in Figure S2.

### 2.6. Cell Depletion

For T-cell depletion, 500 µg anti-mouse CD8 antibodies (clone: 2.43) or anti-mouse CD4 (clone: GK1.5) were injected i.p. into each mouse at 1 day before, and 15 and 30 days after PyNL infection. We confirmed the depletion of CD4 and CD8 T cells by using blood samples analyzed with flow cytometry (AF488-conjugated anti-CD4 (clone: GK1.5), PE-Cy7-conjugated anti-CD8a (clone: 53-6.7), PE-conjugated anti-CD3 (clone: 17A2). Usually, over 99% of CD4^+^ or CD8^+^ T cells were depleted in peripheral blood 24 h after injection. If we find the un-successful depletion, we omitted the data from experiments. For macrophage depletion, mice were injected intravenously (i.v.) with clodronate (Sigma) liposome (1.5 mg clodronate in suspension of 300 μL liposomes) at 3 and 9 days after PyNL infection. We also confirmed the depletion of macrophages in spleen with flow cytometry using PE-conjugated anti-CD11b and PE-Cy7-conjugated anti-F4/80 (clone: BM8.1) when we determined the dose of clodronate liposome in the preliminary examination. Usually, around 90–95% of macrophages in spleen were depleted 24 h after injection when we used the above dose of clodronate liposome.

### 2.7. Prime–Boost Live Vaccination and Cell Transfer Experiments

The mice were primarily infected with PyNL (50,000 pRBCs) at day −90 to −48 and boost immunized with same strain (50,000 pRBCs) at day −8. Normally the peritoneal LN contains only 1–4 × 10^6^ cells per immunized mouse, which is less than 1% of total splenocytes, so we performed a combination of experiments requiring some differences in timing of prime vaccination. Mice were then sacrificed and immunized spleen cells or immunized peritoneal LN cells were collected and single cell suspension was obtained by crushing between the frosted sterile glass slides, RBC were hemolyzed with ACK buffer (NH_4_Cl 8024 mg/l, KHCO_3_ 1001 mg/l, EDTA Na_2_·2H_2_O 3.722 mg/l) and washed 2 times with RPMI1640 plus 10% FCS medium, then cell debris was removed by passing through the filter (50 µm) and then 4 × 10^6^ purified cells were transferred i.v. to recipient mice on day −1. On day 0, recipient mice were infected with PyL (50,000 pRBCs).

### 2.8. Statistical Analysis

The Mann–Whitney U-test was used for statistical analysis between two sets of data. A *p*-value of *p* < 0.05 was considered to be statistically significant. Significant differences in survival were tested with a Gehan–Breslow–Wilcoxon test. GraphPad Prism version 8.0 (GraphPad Software Inc., San Diego, CA, USA) was used in the above analysis.

## 3. Results

### 3.1. Blood-Stage PyNL Infection as a Whole Organism Vaccine

**Figure 1:**
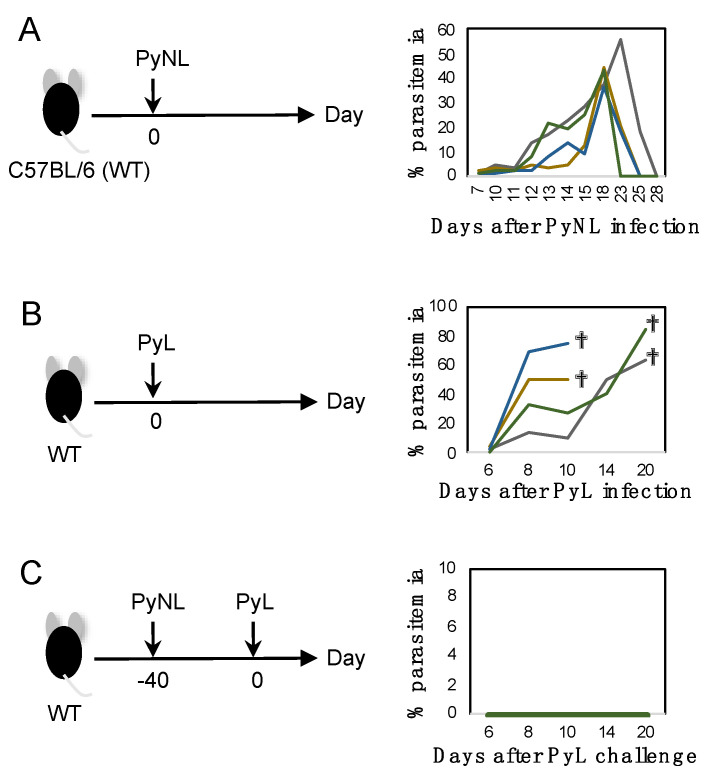
*Plasmodium yoelii* 17XNL (PyNL) infection as a naturally attenuated blood-stage parasite vaccine. Each line indicates parasitemia of one mouse. (**A**) Study protocol of blood-stage PyNL infection (left panel) and changes of parasitemia of PyNL infected mice (right panel). Parasitized red blood cells (RBCs) (50,000 pRBCs) were inoculated i.p. into C57BL/6 mice on day 0. Typically, all mice controlled the infection within 1 month. N = 4. (**B**) Blood-stage PyL (lethal) infection. N = 4. (**C**) PyNL recovered mice were challenged with *Plasmodium yoelii* 17XL (PyL). N = 4.

At the beginning of the experiment, we confirmed the course of infection of blood-stage PyNL in C57BL/6 (WT) mice (Figure 1A left). WT mice were infected with PyNL (5000 pRBC mixed stage of blood-stage parasites) by i.p. injection. Usually, PyNL infection was non-lethal and parasitemia increased to a peak at day 15–23 post-infection of roughly 40% parasitemia. Parasites were then eliminated by the host immune system within 1 month (Figure 1A). Blood-stage parasites parasitize RBC and when merozoites exit infected RBC, the host cells are destroyed. Thus, PyNL induced anemia, as we reported previously [25]. PyL infection (5000 pRBC mixed stage of blood-stage parasites) by i.p. injection was lethal and parasitemia increased to over 50% (Figure 1B). When we challenged mice that recovered from PyNL infection with the cross-strain PyL, which is a lethal strain in naïve WT mice, PyNL-recovered mice showed complete protection against PyL; we did not see emergence of any parasites (Figure 1C). Thus, blood-stage PyNL can be used as a cross-strain protective whole organism vaccine (Figure 1C, Table 1).

### 3.2. Cross-Strain Protection by Blood-Stage PyNL Infection in Immunodeficient Mice

Notably there were a few cases in which the usually non-lethal PyNL killed the WT mice (2 out of 60), indicating the risk associated with live naturally attenuated vaccine when we performed large-scale experiments (Table 1). Here we considered the application of the blood-stage parasite as a vaccine in humans, where the phenotype of the parasite must be stable and must always remain attenuated. Therefore, it is vital to understand the risk of the whole-organism vaccine in different recipients. We also need to know the susceptibility of the vaccine parasite strain to anti-malaria drugs to control an unpredictable infection course if necessary.

First, we considered the administration of our blood-stage naturally attenuated parasite vaccine in immunocompromised individuals. To model this scenario, we immunized several immunodeficient mice, either immune cell depleted or with mutated/knocked out immune molecules, with PyNL (Table 1 and [26,28,29]). There were three expected phenotypes based on the requirement for controlling PyNL infection; 1, if the molecules or cells were essential for protection, all PyNL-infected mice would die. Macrophage depleted and IFN-gamma receptor KO mice fitted this model because all infected mice succumbed to infection. 2, if the molecules or cells were important for protection, some mice would die while others recovered. T cell (CD4 or CD8) depleted, Fas-FasL mutant (Gld or Lpr) and perforin KO mice fitted this model. 3, if the molecules were less important, all mice would recover from PyNL infection. IL-17A KO and LMP7 (immuno proteasome subunit) KO mice fell into this model. We then tested the efficacy of the vaccine against PyL. Once the PyNL infection was controlled, all recovered immunodeficient mice demonstrated sterile cross-strain protection against PyL, similar to WT animals. These results indicate that the whole-organism vaccine may be applicable to some immunodeficient individuals. While, if we do large-scale administration, similar concern as WT mice, which is unpredictable may also occur in KO mice. Thus, we need to carefully decide the application for immunodeficient individuals.

Second, we tested an anti-malaria drug, Art, in our model (Figure S1 left). We administered Art to blood-stage PyNL-infected mice from day 10 to 15 (64 mg/kg body weight/day). The parasitemia peaked on day 10 at around 1–5% before sharply declining (Figure S1 right). Without anti-malaria drug treatment, the parasitemia increased until peak parasitemia was reached at around day 15–23, thus indicating that Art treatment was effective in our vaccine model, and successfully eliminated the parasite.

### 3.3. PyNL Infection Induced Splenomegaly, non-Reversible Changes in the Spleen and Expansion of the Peritoneal LN

**Figure 2:**
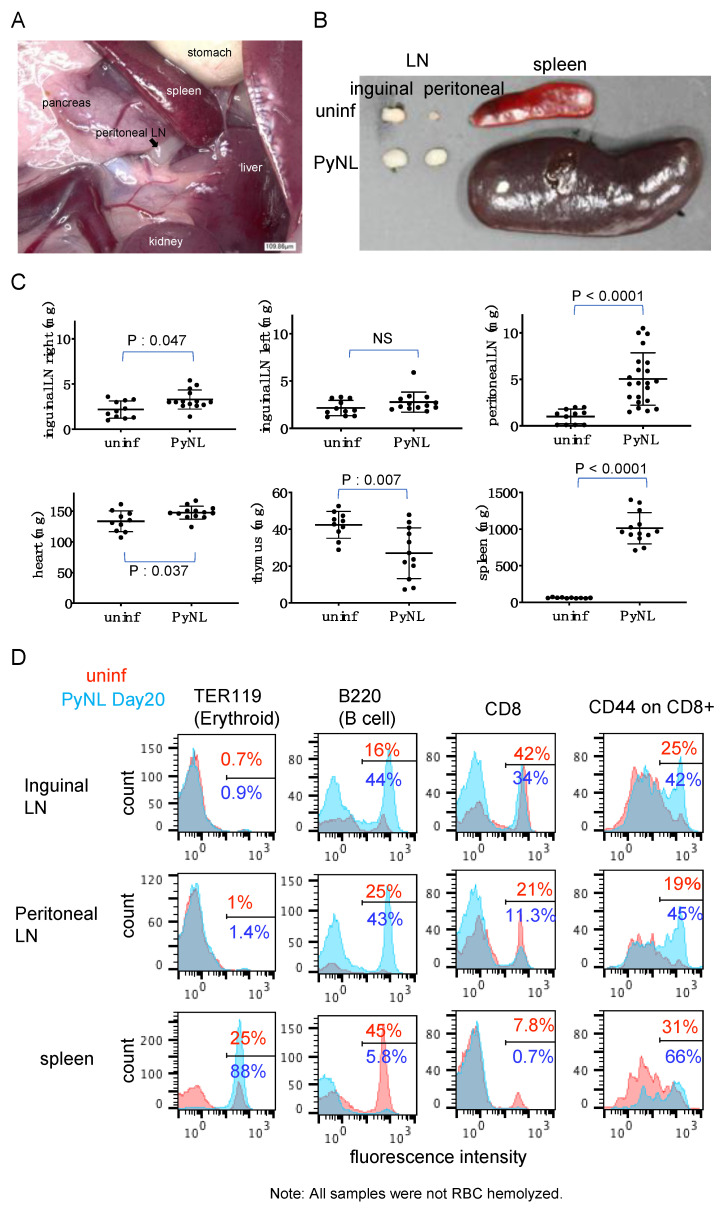
Changes in organ weight and cell proportion following vaccination with blood-stage PyNL. (**A**) Dissected mouse abdomen after PyNL infection. (**B**) Comparison of size and color of spleen and lymph node (LN) between the uninfected mouse (uninf) and PyNL-infected mouse at day 20 post-infection. (**C**) Changes in organ weight; inguinal LN (left and right), peritoneal LN, heart, thymus and spleen were assessed (week 3–4 after infection). Each symbol represents one mouse (N = 11–22). Bars indicate mean ± SD. NS: not significant. (**D**) Flow cytometry analysis of LN and spleen (RBC were not lysed). Overlaid histogram shows uninfected animals (red) and PyNL-infected mice (blue) at day 20. Numbers indicate average (N = 3 mice) proportion of the positive population (erythroid, B, CD8, CD44 in CD8^+^ cells) within gated cells. Gating strategy is shown in Figure S2.

Splenomegaly and hepatomegaly are unique features of malaria infection [1]. To re-confirm the effect of PyNL infection at the tissue level, we sacrificed mice at 3–4 weeks after inoculation and measured the LNs (inguinal and peritoneal), heart, thymus and spleen weight (Figure 2). The peritoneal LN is located near the spleen and liver (Figure 2A).

Spleen weight was dramatically increased, about 50-fold and its color turned into dark red or brown, by infection. The peritoneal LN in PyNL-infected mice was approximately five-fold bigger than in uninfected mice. Usually, the peritoneal LN is so small that sometimes it cannot be found. Thus indeed, the increase might be bigger than five-fold (Figure 2B,C). Heart and the right side of the inguinal LN were also enlarged significantly by infection, but to a lesser extent than the spleen or peritoneal LN. Conversely, the thymus was shrunken (Figure 2C).

Such changes in organ size indicate occurrence of cellular events, and so we chose to further investigate the spleen and LN to understand their role (Figure 2D). In order to analysis of all cells in the organ, this time RBCs were not lysed. Erythroid cells including mature RBCs, reticulocytes, and erythroblasts express TER119 on their surface. Only a few erythroid cells were found in both the inguinal and peritoneal LN with and without PyNL infection (Figure 2D). However, in the spleen 25% of cells were erythroid cells in uninfected mice, but this cell population was dramatically increased to 88% at day 20 post-infection. In rodents, extramedullary hematopoiesis is continuously ongoing in the spleen and it is reasonable that when mice become infected with the malaria parasite, they produce RBC to cover the losses inflicted by the parasite.

**Figure 3:**
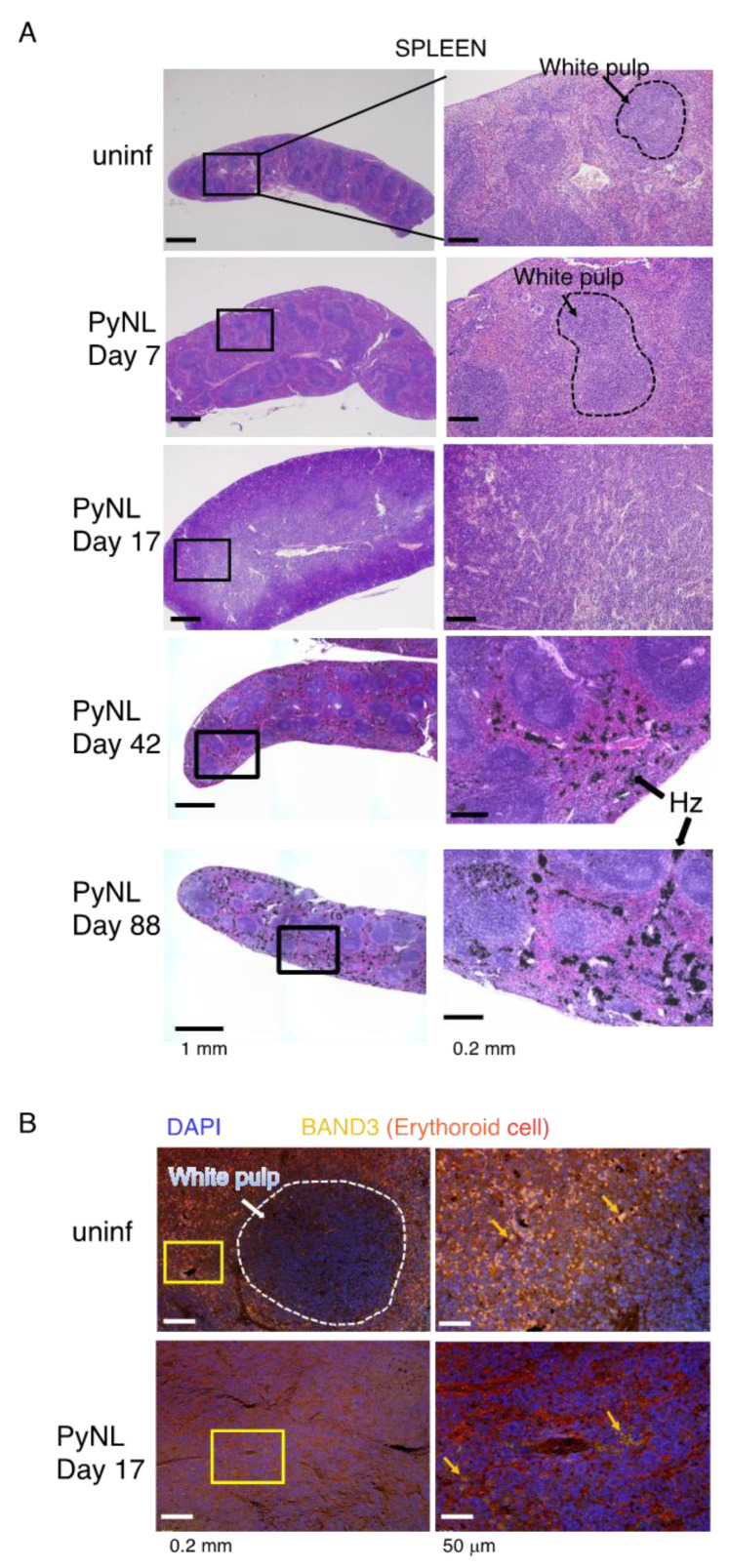
PyNL (blood-stage) infection changes the structure of the spleen. A typical white pulp region in uninfected (uninf) and day 7 PyNL-infected spleens are surrounded by a dashed line. Right panels show enlarged versions of indicated area in the left panel. (**A**) H&E-stained spleens of uninfected and PyNL-infected mice at day 7, day 17 (around peak parasitemia), day 42 (recovered) and day 88 (recovered) are shown. Hemozoin (HZ; black color; indicated by black arrow) was seen in red pulp at day 42 and day 88 post-infection. (**B**) Immuno-staining for BAND3 (erythroid cells; yellow, red, orange) and DAPI (nucleus; blue). Orange arrows indicate examples of positive cells.

We further confirmed the architecture of the spleen by histology (Figure 3). We could clearly distinguish the white pulp and red pulp in uninfected mice based on H&E staining, while this structure was gradually re-constituted by PyNL infection. At day 17, around the time of peak parasitemia, white pulp and red pulp were hardly distinguishable. In other words, the red pulp region was expanded to accommodate massive erythrocyte production, which was consistent with the flow cytometry data (Figure 2C left). Again, we assessed the immunohistostaining with BAND3 [36], which is a marker for erythroid cells (Figure 3B). Consistent with the H&E staining, the white pulp and red pulp were distinguishable in uninfected spleens, but on day 17, BAND3 positive cells were detected throughout the spleen. Additionally, we observed BAND3 positive, DAPI positive nucleated erythroblasts. This result was consistent with our previous finding of expansion of erythroblasts in PyNL-infected mouse spleen or bone marrow [25].

We then followed up the histology after recovery from infection. On day 42, we could again see clear white pulp and red pulp regions, and we frequently detected black entities, which were malaria pigment hemozoins. Notably, hemozoins remained detectable at day 88 post-infection (Figure 3A bottom).

### 3.4. PyNL Induced Activation of Immune Cells in Peritoneal LN but Did not Confer Protection

Next we examined immune cells (Figure 2C) from gated populations based on data presented in Figure S2. When we compare the proportion of B cells in uninfected mice between spleen and LN, B cells dominate the splenic population at around 45%, but not in the LN (16%, inguinal; 25%, peritoneal). The proportion of B cells in PyNL-infected mice was increased to greater than 40% in both LNs. Meanwhile, due to the massive production of erythroid cells in the infected spleen, splenic B cells represented only 5.8% of the cellular population in the spleen following infection, compared with 45% in uninfected animals. Somehow, the proportion of CD8 cells in uninfected mice was different between the inguinal (42%) and peritoneal LNs (21%), both of which were decreased in infected mice due to the expansion of B cells (34%, inguinal; 11.3%, peritoneal). When we account for cell numbers, even if the proportion of CD8 cells was decreased in the peritoneal LN of infected mice, the total cell number (not only for the CD8 cells but the total cells in the peritoneal LN) was increased to five-fold that of uninfected mice (uninf: 3 × 10^5^ cells vs inf: 15 × 10^5^ cells). Indeed, the numbers of CD8 cells in infected peritoneal LNs were increased 2.5-fold compared with uninfected mice (uninf: 6.3 × 10^4^ cells vs inf: 16.5 × 10^4^ cells). This differed from the inguinal LN. The proportion of CD8 cells in infected spleen was reduced compared with uninfected mice, again because of enhanced erythroid cell production. We then gated on CD8 cells and analyzed the expression of CD44, which is a marker of activation or memory phenotype [37]. In all organs we analyzed, the ratio of CD44 expression on CD8-positive cells was increased following infection.

**Figure 4:**
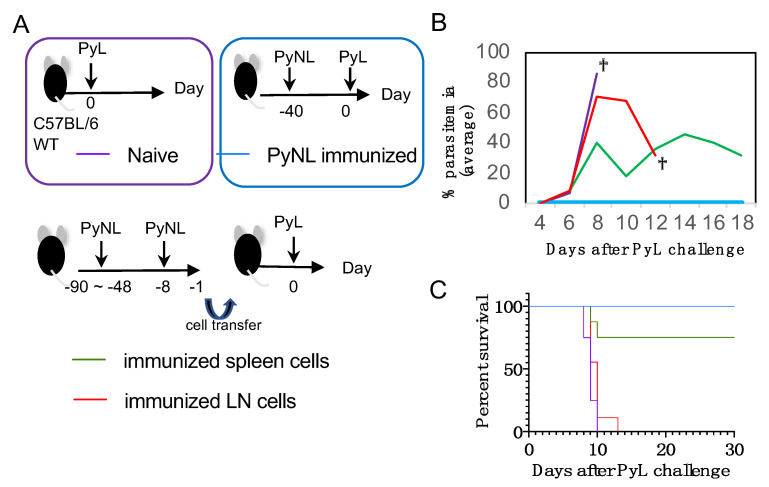
Immunized spleen cells confer protection but immunized peritoneal LN cells did not. (**A**) Experiment protocol. Four groups were challenged with lethal strain PyL at day 0 of the experiment: naïve C57BL/6 mice (upper left: purple line); PyNL immunized mice on day −40 (upper right: blue line); PyNL primed at day −90 to −48, boosted at day −8 then immunized spleen cells (RBC lysed; 4 × 10^6^ cells: green line, 75% of recipient mice controlled the parasitemia) or immunized peritoneal LN cells (4 × 10^6^ cells: red line) were transferred to recipient mice on day −1. (**B**) Average parasitemia and (**C**) survival curve (N = 8 in each group from two pooled independent experiments). Dagger indicates death.

This result inspired us to examine the possibility that the peritoneal LN has immunological memory to confer protection against PyL challenge. Owing to the location and size of peritoneal LNs, we were not able to extract peritoneal LNs, but instead set up a cell transfer experiment. Because we know that the prime and boost vaccination regimen enhances immunological memory, we performed prime-boost vaccination as shown in Figure 4A (bottom). Immunized animals were sacrificed and single cell suspensions of peritoneal LN or RBC-lysed splenocytes (4 × 10^6^ cells) were adoptively transferred into WT recipient mice, before challenge the next day with PyL. PyL-infected unvaccinated mice died within 1 week due to hyperparasitemia and severe anemia (Figure 4B). PyNL-vaccinated mice showed sterile protection against PyL. Some mice transferred with immunized splenocyte suspension controlled the infection (75%), although, unexpectedly, transferred with immunized peritoneal LN cells had no effect on survival (Figure 4C). These results indicate that resident peritoneal LN cells do not contribute to conferring protection to recipient mice.

### 3.5. Limited Cross-Species Protection by PyNL Infection

**Figure 5:**
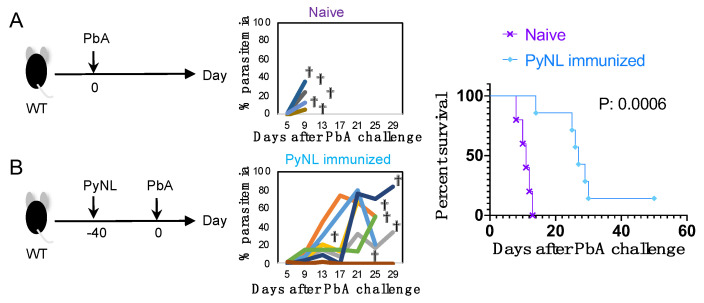
Limited cross-species protection against *Plasmodium berghei* ANKA (PbA) challenge following PyNL infection. Left panels show experiment protocol. Each group was challenged with blood-stage PbA on day 0. Center panels show parasitemia of each mouse. Each line indicates one mouse. Right panels show survival curves (N = 5–7). Two groups were compared. All data were pooled from two independent experiments. (**A**) Naïve mice (N = 5). (**B**) blood-stage PyNL immunized mice (N = 7). One mouse attained a maximum of 1.7% parasitemia at day 14 after PbA infection and cleared the parasite at day 20, while the remaining six animals succumbed to infection. Dagger indicates death.

Finally, we evaluated the efficacy of PyNL infection against challenge with a different species, PbA (Figure 5). PbA infection was lethal within 2 weeks in naïve WT mice and caused cerebral malaria (Figure 5A). PyNL infection prolonged survival (*p* = 0.0006) and one out of seven mice recovered from infection without recurrences (Figure 5B). Compared with cross-strain protection, cross-species protection was much less effective and did not induce sterile protection.

## 4. Discussion

We have previously analyzed the protective role of T cells or immunological molecules, or both, against blood stage malaria [26,28,29], and found CD4 and CD8 T cells to be important but not essential for protection against primary infection with blood-stage PyNL (Table 1) [26]. T cells and B cells possess immunological memory so induction of pathogen-specific memory cells is essential for sterile protection against malaria. However, macrophages are always essential in primary and challenge infections (Table 1) [26]. Here we have described the potential and limitations of cross-protective vaccination against malaria using blood-stage naturally attenuated parasites. Our results indicate that cross-strain protection is possible but achieving cross-species protection is difficult. The histopathological changes presented during the vaccination process may trigger several adverse reactions and signs/symptoms not covered in this study.

Genetic diversity of *Plasmodium* spp. [38,39,40] is one of the reasons for the challenges involved in generation of a cross-species vaccine. To overcome such cross-species differences, many researchers have generated the rodent malaria parasite, which expresses the CSP of the avian or human malaria parasite [41,42,43]. Some of these chimeric parasites produced salivary gland sporozoites (an infectious type of parasite) to similar levels as WT parasites, and anti-chimeric parasite immune responses were observed. This indicates that it is possible to develop a cross-species protective vaccine. Then, Marin-Mogollon et al. tried to make a recombinant Pf that expressed *P. vivax* CSP [44], to develop a cross-protective human malaria vaccine. They successfully generated the blood-stage recombinant parasite (asexual and sexual); however, unfortunately this recombinant parasite did not produce hemocoel or salivary gland sporozoites in the mosquito stage. Artificial protein GFP-, mCherry- and luciferase-expressing Pf was generated and was able to develop all stages of the parasite [45,46] thus indicating that Pf at least allows the expression of exogenous protein but *P. vivax* CSP may inhibit original Pf function.

Another concern of cross-species protective vaccine is the role of immune cells in malaria infection. When we depleted CD8^+^ T cells in primary infection with blood-stage PyNL, half of the mice died (Table 1) but in PbA infection, survival was improved [27]. Thus, the role of CD8+ T cells in primary infection with PyNL is positive (protective) for the host but negative (pathological) in PbA infection. Our data suggest that PyNL infection prolonged survival following challenge with PbA and almost no cerebral malaria developed in immunized WT mice. Thus, PyNL infection could induce a protective response rather than pathology following PbA challenge infection.

Splenomegaly is seen in malarial rodents and humans [31]. Our data show that the increase in volume is mainly due to the production of erythroid cells but not immune cells. In our previous report, spleen and bone marrow contain erythroblasts, which are the precursor cells of RBCs, and the number of erythroblasts becomes increased following infection with PyNL [25]. This is quite reasonable because peak parasitemia of PyNL can reach greater than 50% and the blood-stage parasite cycle is completed within 24 h, thus indicating that PyNL lyses 50% of RBCs within 1 day. If hematopoiesis is not sufficient, parasitemia becomes lethal. The bone marrow is also capable of hematopoiesis in such an emergency, but the volume of space within the bone limits the RBC-producing capacity while there is no limitation to the spleen in terms of volume. Thus, the spleen has at least three roles for protection against blood-stage malaria: first is its biological filter function, supported by macrophages; second is the storage of immunological memory T and B cells; and third is its function as factory for RBC hematopoiesis [30,31,47].

We found that peritoneal LN were also enlarged. We speculate that malaria parasite antigen-presentation or encounter with malaria parasites (antigens) in peritoneal LN might be more frequently happen than the other LNs. Because peritoneal LNs locates in between spleen and liver, where accumulate and eliminate the malaria parasite by macrophages. However, we have one concern whether the enlargement of peritoneal LN in infected mice is due to the intraperitoneal infection of blood-stage malaria parasite method or not. This peritoneal LN from malaria infected mice contained activated or memory-like marker expressing cells. However, when we adoptively transferred these cells to mice followed by challenge, they offered no protection. Thus, LN may not be as important in blood-stage malaria infection. Further experiments are required to explain a mechanism that enlargement of peritoneal LN and it does not contribute to protection. For example, infection to alymphoplasia (Aly/Aly) mice would provide some insight for understanding of the importance of LN in blood-stage malaria protection [48]. Unlike the blood-stage, sporozoites are trapped in skin draining LN and seem to be related to protection [49].

We also found hemozoins in the spleen. Hemozoin has immunostimulatory function [50] and remaining hemozoin causes serious side effects. Frita et al. also reported hemozoin is found in the liver for a long time, up to day 196 after infection [51]. When we treated with Art, the parasite-eliminated mice did not need to produce excessive RBC, as seen in untreated mice during infection; indeed, spleen weight was approximately 50% of that in an untreated mouse (data not shown). Additionally, Art-treated mice should have decreased parasite burden, resulting in decreased splenomegaly and we speculate that hemozoin may be decreased in Art-treated mice. Lin et al. developed a recombinant parasite lacking the enzyme gene and protein related to hemoglobin digestion, and this mutant did not produce hemozoin [52]. This parasite gene seemed to be a good candidate of a parental parasite to generate a blood-stage human GAP, but the mutant parasite was resistant to chloroquine, which highlights the importance of selecting the gene to be deleted.

## 5. Conclusions

We have demonstrated the potential and limitations of cross-protective vaccine against malaria by blood-stage naturally attenuated parasite.

Currently, there is no rodent or human blood-stage malaria GAP, the generation of which has multiple difficulties, for example, there is no good long-term in vitro culture system for rodent malaria parasites for proof of concept. We have a Pf culture system [53], but an attenuated parasite means that the parasite must die in the host blood stream. We therefore need some technological innovation, so that the parasite is only viable under the specific conditions of in vitro culture but dies when injected into humans. Hopefully, these blood-stage attenuated parasite or GAPs have no adverse side effects. Additionally, if a multi-species antigen-expressing GAP is developed, not only cross-strain but also cross-species immunity will be conferred to humans, which will undoubtedly contribute greatly to global health. Recently, Stanisic et al. reported a new method for making blood-stage attenuated parasites for the vaccine. Which is chemically attenuation by using cyclopropylpyrolloindole analogue, tafuramycin-A, in vitro. In their clinical study, they confirmed the safety and effectiveness of induction of Pf specific immune response in the volunteer by chemically attenuation vaccine [54]. This vaccine might be the next hope of a blood-stage malaria vaccine.

## Figures and Tables

**Table 1:** Cross-strain protection by PyNL infection followed by PyL challenge in immunodeficient mice.

Mouse	N (7–60)	Cure rate of PyNL Infection	Result of PyL Challenge to PyNL Immunized
C57BL/6 (WT)	60	97%	Complete protection
CD4 depletion	20	50%	Complete protection
CD8 depletion	56	50%	Complete protection
Macrophage depletion	10	All died.	Not tested.
IL-17A KO	9	100%	Complete protection
LMP7 (immuno proteasome subunit) KO	16	100%	Complete protection
MFGE-8 KO	7	100%	Complete protection
Lpr (Fas mutant)	20	85%	Complete protection
Perforin KO	12	50%	Complete protection
Gld (Fas L mutant)	25	48%	Complete protection
IFN-gamma Receptor KO	7	All died.	Not tested.

## Supplementary Materials

The following are available online at https://www.mdpi.com/2076-393X/8/3/375/s1. Figure S1. Treatment with anti-malaria drug artesunate (Art). Left panel: protocol of experiment. Infection with 50,000 parasitized red blood cells (pRBCs) were performed on day 0, Art (64 mg/kg body weight/day) was administered i.p. from day 10 to day 15 post-infection. Right panel: change of parasitemia. Each line indicates one mouse (N = 6). Figure S2. Flow cytometry gating strategy. Organs from uninfected or PyNL-infected mice were harvested at day 20 and dissociated to single cell suspensions, Fc blocked then stained with indicated the antibody cocktails. Surface staining dead cells were then stained with zombie dye, as described in the Materials and Methods. The flow cytometry analysis gating strategy is shown. Spleen cells without RBC lysis are used as an example, the same gate was used for all samples. (**A**) FSC vs SSC plot. Cell debris is excluded from analysis. (**B**) Gated populations in A were further gated for single cells by FSC-A vs FSC-H. (**C**) Gated populations in B were further gated for live cells, then used for analysis in Figure 2D.
