# Supplementary material for: Potential and Limitations of Cross-Protective Vaccine against Malaria by Blood-Stage Naturally Attenuated Parasite
Source: Vaccines (Basel). 2020 Jul 11;8(3):375. doi: 10.3390/vaccines8030375 (PMC7564742; doi:10.3390/vaccines8030375)
Supplement: Supplementary file 1 [file vaccines-08-00375-s001.pdf]

vaccines MDPI

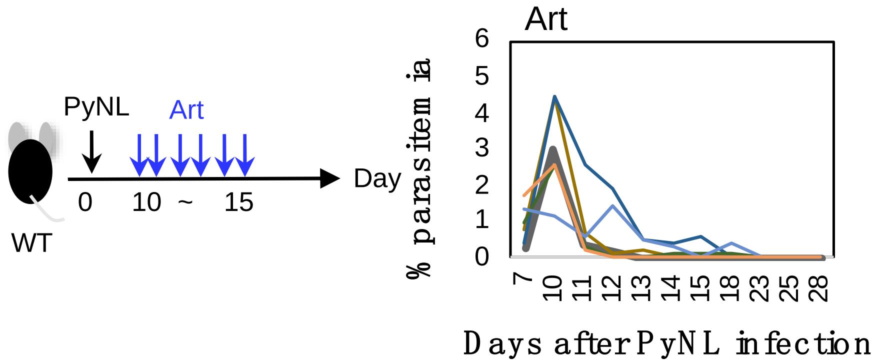

Figure S1. Treatment with anti-malaria drug artesunate (Art). Left panel: protocol of experiment. Infection with 50,000 parasitized red blood cells (pRBCs) were performed on day 0, Art (64 mg/kg body weight/day) was administered i.p. from day 10 to day 15 post-infection. Right panel: change of parasitemia. Each line indicates one mouse (N = 6).

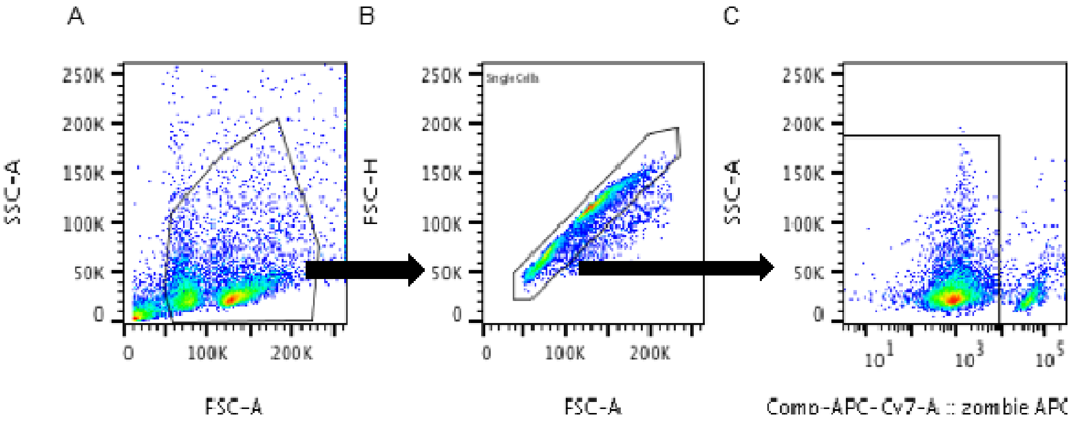

Figure S2. Flow cytometry gating strategy. Organs from uninfected or PyNL-infected mice were harvested at day 20 and dissociated to single cell suspensions, Fc blocked then stained with indicated the antibody cocktails. Surface staining dead cells were then stained with zombie dye, as described in the Materials and Methods. The flow cytometry analysis gating strategy is shown. Spleen cells without RBC lysis are used as an example, the same gate was used for all samples. (**A**) FSC vs SSC plot. Cell debris is excluded from analysis. (**B**) Gated populations in A were further gated for single cells by FSC-A vs FSC-H. (**C**) Gated populations in B were further gated for live cells, then used for analysis in Figure 2D.
